# Bioinspired Photonic Materials from Cellulose: Fabrication, Optical Analysis, and Applications

**DOI:** 10.1021/accountsmr.3c00019

**Published:** 2023-05-24

**Authors:** Richard M. Parker, Thomas G. Parton, Chun Lam Clement Chan, Mélanie M. Bay, Bruno Frka-Petesic, Silvia Vignolini

**Affiliations:** Yusuf Hamied Department of Chemistry, University of Cambridge, Lensfield Road, Cambridge, CB2 1EW, United Kingdom

## Abstract

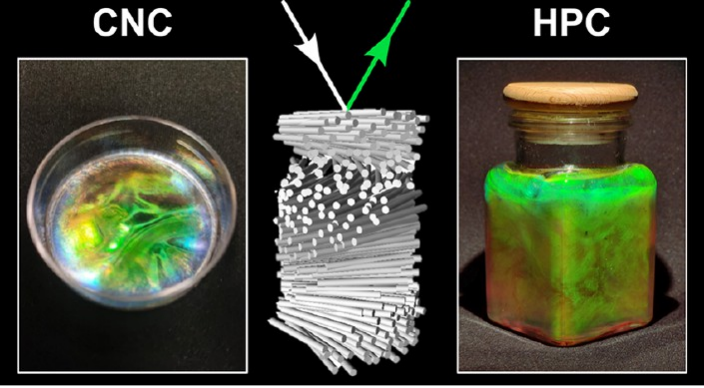

Polysaccharides are a class of biopolymers that are widely exploited in living organisms for a diversity of applications, ranging from structural reinforcement to energy storage. Among the numerous types of polysaccharides found in the natural world, cellulose is the most abundant and widespread, as it is found in virtually all plants. Cellulose is typically organized into nanoscale crystalline fibrils within the cell wall to give structural integrity to plant tissue. However, in several species, such fibrils are organized into helicoidal nanostructures with a periodicity comparable to visible light (i.e., in the range 250–450 nm), resulting in structural coloration. As such, when taking bioinspiration as a design principle, it is clear that helicoidal cellulose architectures are a promising approach to developing sustainable photonic materials.

Different forms of cellulose-derived materials have been shown to produce structural color by exploiting self-assembly processes. For example, crystalline nanoparticles of cellulose can be extracted from natural sources, such as cotton or wood, by strong acid hydrolysis. Such “cellulose nanocrystals” (CNCs) have been shown to form colloidal suspensions in water that can spontaneously self-organize into a cholesteric liquid crystal phase, mimicking the natural helicoidal architecture. Upon drying, this nanoscale ordering can be retained into the solid state, enabling the specific reflection of visible light. Using this approach, colors from across the entire visible spectrum can be produced, alongside striking visual effects such as iridescence or a metallic shine. Similarly, polymeric cellulose derivatives can also organize into a cholesteric liquid crystal. In particular, edible hydroxypropyl cellulose (HPC) is known to produce colorful mesophases at high concentrations in water (ca. 60–70 wt %). This solution state behavior allows for interesting visual effects such as mechanochromism (enabling its use in low-cost colorimetric pressure or strain sensors), while trapping the structure into the solid state enables the production of structurally colored films, particles and 3D printed objects.

In this article, we summarize the state-of-the-art for CNC and HPC-based photonic materials, encompassing the underlying self-assembly processes, strategies to design their photonic response, and current approaches to translate this burgeoning green technology toward commercial application in a wide range of sectors, from packaging to cosmetics and food. This overview is supported by a summary of the analytical techniques required to characterize these photonic materials and approaches to model their optical response. Finally, we present several unresolved scientific questions and outstanding technical challenges that the wider community should seek to address to develop these sustainable photonic materials.

## 1 Drawing Inspiration from Nature

When developing new functional materials, one can take inspiration from nature, where a limited selection of simple components can be assembled into various hierarchical structures for a wide range of roles.^1^ This concept is well exemplified by plants, where diverse functions can be achieved by relatively small variations in polysaccharide composition and morphology. Among these polysaccharides, cellulose (a linear homopolymer of β-d-glucopyranose) is the most prevalent and is principally employed to give structural integrity to the plant cell wall. To achieve this, cellulose is hierarchically organized into crystalline nanoscale fibrils, which can then be combined or assembled into a variety of larger scale architectures that confer different functionalities.

**Figure 1:**
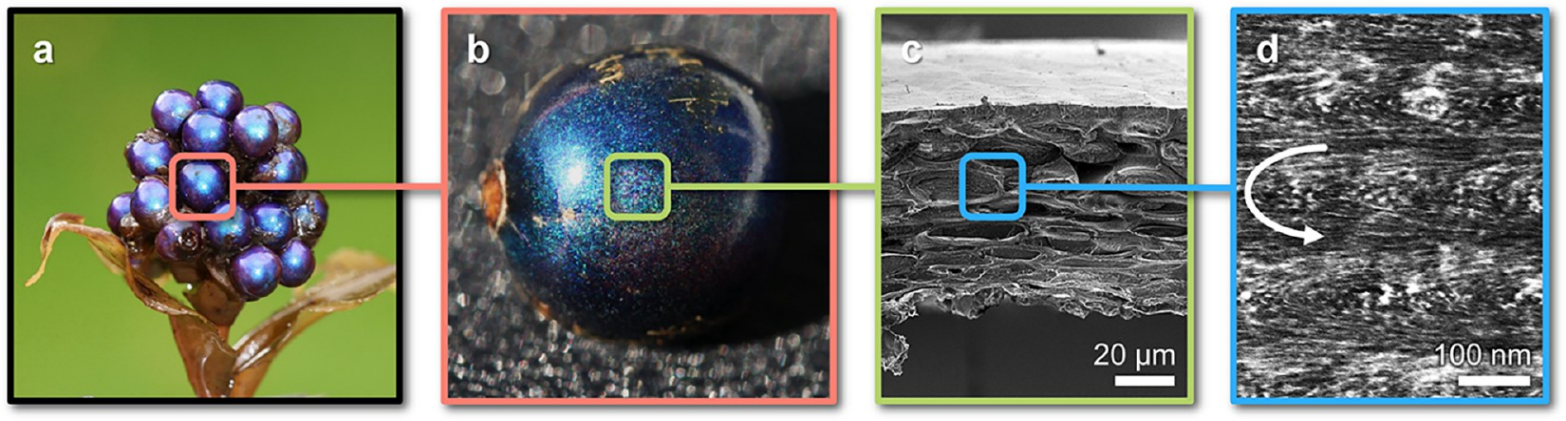
The vibrant blue color of the Pollia condensata fruit (Ø ≈ 5 mm), as shown in (a,b), arises from the helicoidal arrangement of cellulose fibrils in the cell walls of the epicarp, as visualized by (c) cross-sectional scanning electron microscopy and (d) transmission electron microscopy. Photographs (a,b) adapted with permission from ref (2). Copyright 2012 The Authors. EM images (c,d) adapted with permission from ref (3). Copyright 2021 The Authors.

One of the more unusual applications of such hierarchical architectures is to achieve coloration, as seen in the striking blue fruits of *Pollia condensata* (Figure 1a,b).^2^ In these plants, this so-called “structural coloration” arises from the helicoidal organization of cellulose microfibrils within the epicarp of the fruit. The microfibrils are locally aligned in planes, but with a twist perpendicular to the alignment axis (Figure 1c,d). When the periodicity (or “pitch”) of this helicoidal structure is comparable to the wavelength of visible light, constructive interference occurs from this otherwise transparent material, resulting in a strong reflection over a narrow range of wavelengths. Moreover, this reflected light can be left or right circularly polarized (LCP or RCP) depending on the handedness of the helicoidal structure. In plants, such structures are almost universally left-handed; however, a small proportion of cells in *Pollia condensata* predominantly reflect RCP light.^2^ This unusual structural inversion is attributed to differences in the cell wall composition, suggesting the importance of hemicelluloses in mediating the interactions between neighboring fibrils.^3^

From the iridescent wings of butterflies to the striking feathers of birds, examples of structural color can be found throughout the natural world as it offers numerous advantages.^4^ For instance, structurally colored materials often appear more vibrant than absorption-based pigments. Moreover, because the reflected wavelength band is dependent on the precise dimensions of the underlying nanostructure, rather than a specific absorption of a dye molecule, these colors can be tuned across the entire visible spectrum and are not subject to bleaching or fading, enabling their long-term stability. As such, it is desirable to replicate these nanostructured materials, especially using more sustainable alternatives to existing mineral or polymer-based pigments.^5^ Cellulose is the most abundant biopolymer on Earth, with over 10^12^ tonnes of biomass estimated to be produced each year.^6^ While it can be extracted from almost any plant, common sources include cotton and wood, due to their relatively high cellulose content and commerical availability. At the same time, there is untapped potential to valorize cellulose from agricultural and food waste and end-of-use textiles as part of a circular economy model. In this article, we present two strategies to reproduce the helicoidal architecture seen in plants via the self-assembly of colloidal and polymeric cellulose derivatives and describe how these approaches can be exploited to produce photonic materials with vibrant structural color.

## 2 Structurally Colored Films via Colloidal Self-Assembly

**Figure 2:**
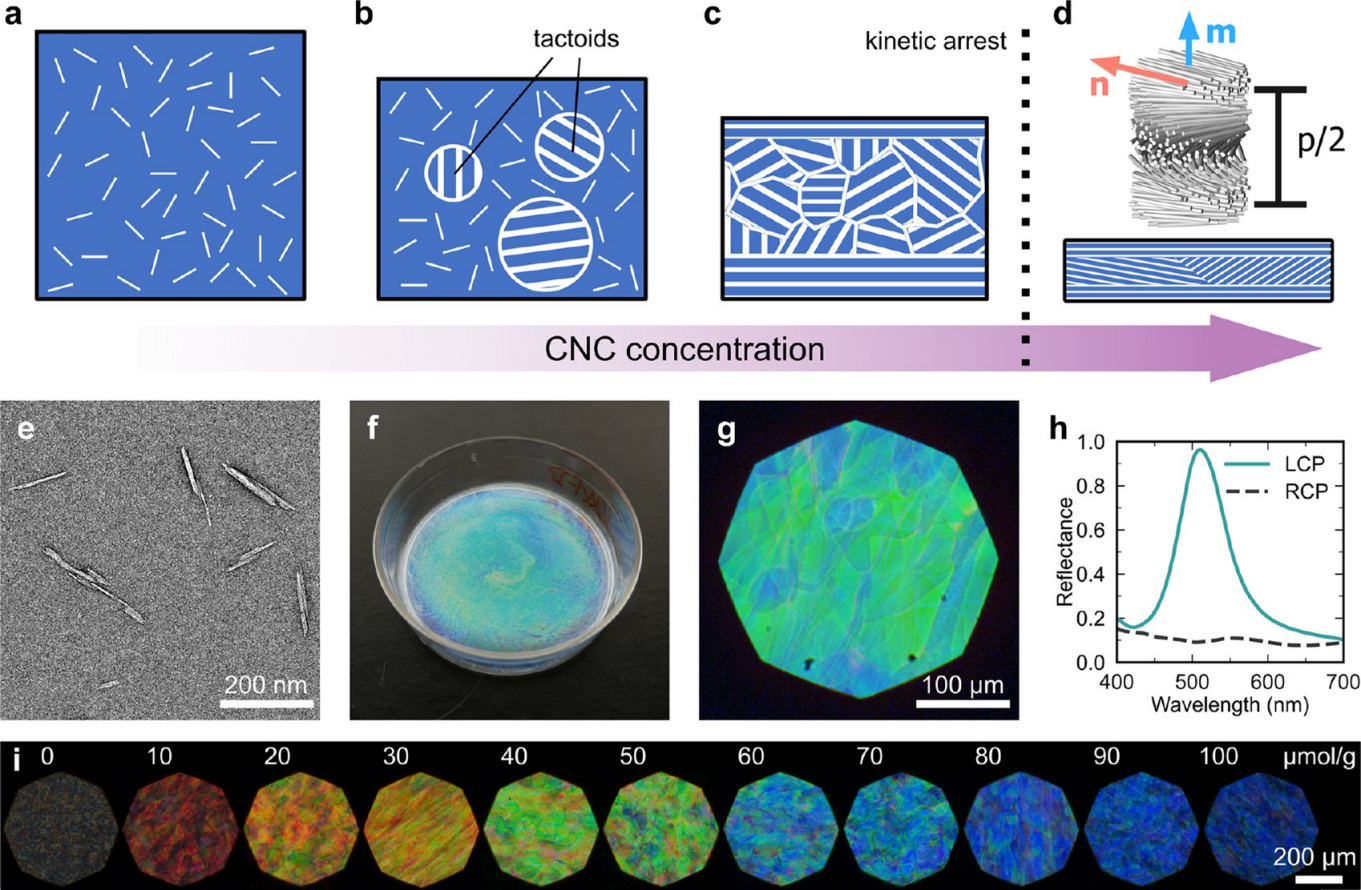
(a–d) Evolution of a CNC suspension upon increasing concentration, from an isotropic state (a) to the emergence of the cholesteric phase as tactoids (not to scale) (b), then a fully cholesteric polydomain structure (c) that undergoes kinetic arrest and dries to form a solid helicoidal photonic film (d). Inset represents cholesteric ordering. Reproduced with permission from ref (11) under CC-BY. Copyright 2016 The Authors. (e) Typical CNC morphology as observed in TEM. Data from ref (12). (f–h) Optical characterization across length-scales: (f) photograph of a photonic film in a 35 mm diameter Petri dish (g) polarized optical micrograph of the same film with (h) corresponding polarized microspectroscopy. Reproduced with permission from ref (13). Copyright 2021 The Authors. (i) Tuning the color of photonic CNC films across the visible spectrum by varying the ratio of added electrolyte (NaCl) per CNC dry mass in units of μmol/g.

Native cellulose fibers from plants are arranged into highly crystalline microfibrils.^7^ As such, by applying a strong acid hydrolysis to natural cellulosic biomass (typically H_2_SO_4_, or HCl followed by TEMPO oxidation), crystalline nanoparticles can be obtained. Such “cellulose nanocrystals”, or simply “CNCs”, are comprised of one or more laterally bound cellulose crystallites. Each CNC typically has an elongated shape, with a width of up to a few tens of nanometers and a length of several hundred nanometers, as measured by atomic force microscopy or transmission electron microscopy (Figure 2e). The *cell*-O-SO_3_H or *cell*-COOH groups grafted during hydrolysis impart a negative surface charge to the CNCs (typically on the order of 10–100 μmol/g), which enables them to form a stable colloidal suspension in water (with typical zeta potential values of |ζ| > 30 mV). However, the precise properties of these irregular, polydisperse nanoparticles are intrinsically linked to both the source and the production conditions, which necessitates detailed characterization of each batch.^8^

Above a threshold concentration, CNCs can spontaneously organize via a nucleation and growth mechanism into a cholesteric liquid crystal, often synonymously referred to as a chiral nematic phase (Figure 2a–d).^9^ In this phase, the nanoparticles locally align along a common direction, defined as the director **n** that spatially rotates in a left-handed helicoidal structure about a helical axis **m** and with a pitch *p*, which is defined by a 360° rotation of the director. Importantly, while at low concentration, the cholesteric pitch is typically on the micron-scale, and it reduces with increasing CNC volume fraction (approximated to a power law of *p* ∝ Φ^–1^). Upon further concentration, the suspension undergoes kinetic arrest, which allows for this ordering to be retained into the solid state, mimicking the natural helicoidal architecture.

Owing to their high crystallinity, CNCs retain the intrinsic birefringence of native cellulose I. As such, when assembled into a cholesteric architecture there is a helicoidal modulation of the refractive index along **m**. Consequently, light interference within this periodic structure leads to intense polarized reflection in a specific wavelength range, as discussed further in Section 4.^10^ Thus, by controlling the self-assembly of a CNC suspension, it is possible to produce an iridescent film with vibrant strucutral color (Figure 2f–h).

### 2.1 Designing a Photonic CNC Film

The most common method to produce a photonic CNC film is to evaporate a dilute aqueous CNC suspension (∼1–3 wt %) within a shallow container, such as a Petri dish, under ambient conditions. However, while this process appears straightforward, there are many parameters that require optimization to achieve an intense photonic response.^14^

The cholesteric pitch is determined by the strength of the chiral interactions between the CNCs and their packing density (i.e., their volume fraction). As such, it is determined by both the intrinsic properties of the CNCs and their surrounding medium. Fundamentally, the cellulose source (e.g., cotton vs wood-pulp) and the production conditions (i.e., hydrolysis and purification) determine the key morphological and electrostatic properties of the CNCs,^15^ which together define the overall parameter space that the suspension can be tuned across. Notably, by exploiting the tendency for biphasic suspensions to fractionate under gravity, CNCs with high aspect ratio can be isolated, which offer both a stronger helical twisting power (leading to smaller pitches)^16^ and an earlier phase separation (potentially extending the self-assembly window, see below).^17^

Once a CNC suspension is selected, there are several formulation parameters that are routinely adjusted to tune the reflected wavelength of the resultant film. For example, the final pitch (and thus the color) can be blue-shifted by weakening the repulsive interactions between CNCs in suspension, either by lowering the surface charge on the CNCs (e.g., heat-induced desulfation^18^) or by screening these charges via addition of an electrolyte (e.g., NaCl, HCl, H_2_SO_4_^10^), as shown in Figure 2i. Conversely, the final color can be red-shifted by (i) reducing the helical twisting power by breaking apart the crystallite bundles that act as colloidal chiral dopants (e.g., via tip sonication^12,19^) or (ii) by the replacement of water with a nonvolatile hydrophilic additive (e.g., glucose,^20^ poly(ethylene glycol)^21^), which prevents complete collapse of the helicoidal nanostructure upon drying (as well as improving film uniformity). Moreover, while these parameters are often considered in simple terms of how they shift the pitch, it is important to note that they frequently influence other aspects of the self-assembly process (such as the viscosity, phase behavior or the onset of kinetic arrest) and thus they typically need to be optimized concertedly to achieve a wide gamut of intense colors.

The complex dynamics of drying a CNC suspension combined with the geometry of the container play a key role in determining the visual appearance of the resultant film. Self-assembly can only occur within the concentration range between the onset of liquid crystal formation and kinetic arrest (Figure 2a–d), where cholesteric droplets (termed “tactoids”) can form, sediment, reorient and finally merge. Maximizing the time spent in this “self-assembly window” enables large domains to form with minimal defects, resulting in greater uniformity of the helicoidal nanostructure (and thus a more vibrant optical response). While this concentration range is specific to the suspension, the duration of self-assembly can be controlled via the initial CNC concentration (assuming fixed suspension volume) and the evaporation rate (which is typically constant for a dish geometry over the majority of the drying process). As such, a common strategy to improve the quality of the photonic response is to extend the overall time taken to dry the suspension into a film.^22^ Alternatively, differential evaporation rates (e.g., via a mask or localized heating) can be exploited to impart color gradients or patterns to the photonic CNC film.^13,23^ The long-range cholesteric order within a drying suspension can also be actively enhanced, either by orienting CNC tactoids using mild magnetic fields (μ_0_*H* ≈ 0.5–1 T)^24^ or by applying orbital shear flow to an isotropic CNC suspension.^25^ Finally, it is important to ensure that the suspension remains pinned to the dish walls during drying, which promotes the uniaxial vertical compression of the suspension after kinetic arrest that is vital to reach the submicron pitch values needed for visible color.^26^

The functionality of photonic CNC films can be further extended by incorporating additives or applying post-treatments. For example, the hydrophilic polymer, hydroxypropyl cellulose (HPC) can be incorporated as a nonvolatile plasticizer, which provides a direct route to tune the photonic response and enhance the flexibility of a CNC film, while maintaining a fully cellulosic composition.^27,28^ Furthermore, hygroscopic additives (e.g., poly(ethylene glycol)) can endow the film with hygrochromic swelling (unlocking application as a humidity sensor),^21^ while doping with achiral luminophores can be used to generate circularly polarized luminescence or to use CNC films as a cholesteric laser.^29^ Alternatively, thermal post-treatment of CNC films leads to desulfation,^18^ which can be exploited to prevent the redispersibility of a CNC film in polar solvents, even water.^13^ Finally, CNCs can be combined with other functional materials, such as elastomers (to achieve a mechanochromic response or shape memory behavior),^30,31^ cross-linkers (to improve film robustness),^32^ or reinforcing nanofibers (which enhance tensile strength and toughness).^33^

An ideal photonic CNC film reflects up to 50% of incident unpolarized light due to its left-handed helicoidal structure, which selectively reflects only the LCP component (as discussed in Section 4). However, this theoretical limit can be overcome by drawing inspiration from the cuticle of the golden scarab beetle *Chrysina resplendens*, where a birefringent layer (that acts as a half-wave plate) between two left-handed helicoidal domains results in strong reflection of both LCP and RCP light.^34^ An analogous trilayer structure has been created by laminating CNC films, with the intermediate layer consisting of either a birefringent polymer (e.g., Nylon sheet)^35^ or a quasi-nematic CNC layer produced by deposition under high shear.^36^ Alternatively, infiltration of a CNC film with a nematic liquid crystal (e.g., 5CB) can also be used to produce this effect, with the total reflection tunable by either temperature variation or an applied AC electric field.^37^

### 2.2 Scalable Deposition Techniques

**Figure 3:**
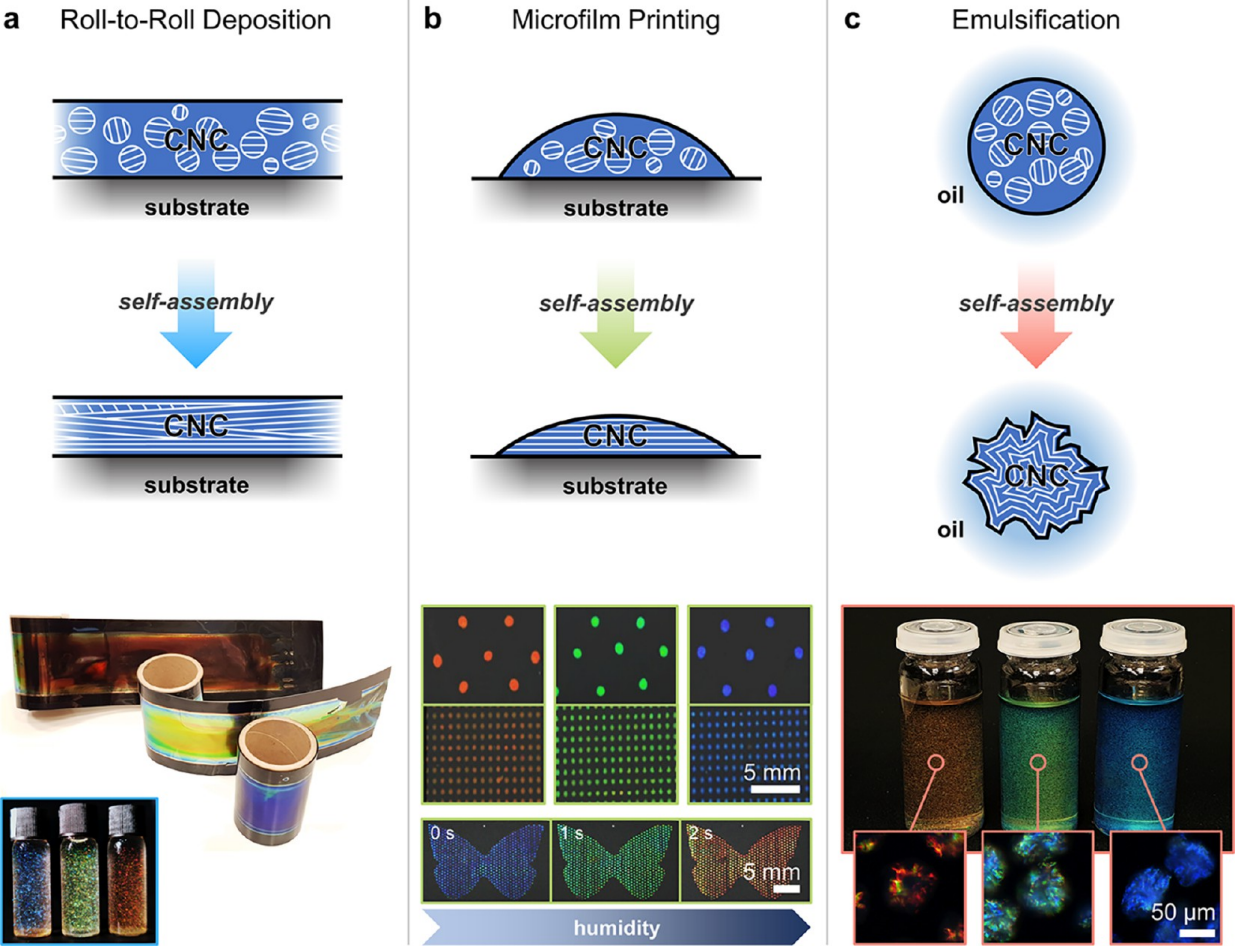
Three proposed pathways to upscale the production of photonic CNC materials: (a) Continuous R2R deposition for large-scale production of films, which can be postprocessed into glitter. Reproduced with permission from ref (13). Copyright 2021 The Authors. (b) Dot-matrix printing of sessile drops to form responsive microfilm arrays. Reproduced with permission from ref (39) under CC-BY. Copyright 2018 The Authors. (c) Confined self-assembly within a microemulsion for direct production of pigments. Reproduced from ref (43) under Creative Commons CC-BY. Copyright 2022 The Authors.

To develop photonic CNC materials toward real-world application as sustainable colorants, there has been a recent drive to move beyond small-scale or batch-based casting processes. For example, while the dimensions of a dish-cast film are typically defined by the geometry of the container (e.g., diameter, Ø < 10 cm), meter-scale films can be continuously cast using Roll-to-Roll (R2R) deposition (Figure 3a). This approach can be used to scale up the production of sustainable effect pigments (e.g., glitter)^13^ or to produce functional laminates for applications such as subambient daytime radiative cooling.^38^

Alternatively, by drawing analogy to dot-matrix printing, arrays of CNC microfilms (Ø < 1 mm) can be used to coat surfaces or even produce images.^39^ In this approach, the CNC suspension is deposited as a sessile drop (i.e., a nanoliter-scale volume of suspension wetting a surface). However, the small size of such drops makes it challenging to control their drying dynamics to produce films with uniform and intense reflection. In the absence of a container, a drop of CNC suspension will spread across a hydrophilic substrate to reach an equilibrium shape, which is governed by a combination of surface tension and substrate chemistry. Upon drying, the greater evaporation rate at the edge of the drop generates an outward radial capillary flow that leads to the accumulation and deposition of CNCs near the contact line.^40^ This so-called “coffee-ring effect” disrupts self-assembly and causes a significant color-shift across the microfilm profile.^41^ To overcome this issue, drops can be laterally confined using a hydrophilic/hydrophobic patterned substrate (minimizing dewetting) and dried under a layer of oil, which suppresses the evaporation gradient that leads to the coffee-ring effect. This results in monodomain microfilms with a near-ideal optical response (Figure 3b).

Lastly, the confinement of a cholesteric CNC phase within a micron-scale spherical droplet allows for hierarchical microparticles to be continuously produced via an emulsion-based process.^42,43^ In this spherical geometry (when Ø ≈ 80–240 μm), planar anchoring of the CNCs at the liquid–liquid interface results in the formation of a monodomain structure with **m** radially aligned (i.e., Frank-Pryce-like ordering).^44^ Although this arrangement is conserved upon drying, the pitch in such CNC microparticles was much larger than expected for the standard film geometry, resulting in no visible coloration.^42^ This is attributed to the isotropic 3D compression specific to a contracting sphere, resulting in the pitch following *p* ∝ Φ^–1/3^ after the point of kinetic arrest (Φ_KA_). As such, to produce visibly colored microparticles (Figure 3c), additional compression can be introduced by exploiting interfacial buckling, which results in a pitch contraction that is more analogous to that of the film geometry (i.e., *p* ∝ Φ^–1/3^ → Φ^–1^).^43^ Interestingly, in contrast to uniaxially aligned CNC films, radially aligned CNC microspheres give rise to angle-independent color under diffuse illumination.

As a final comment, the three fabrication methods described above are already widely used in industry in other contexts with high throughput. For example, film deposition by R2R or continuous inkjet printing can be performed at over 100 m/min, while continuous microdroplet generation by membrane emulsification can be achieved at over 1000 L/h. Comparable rates can in principle be achieved with CNC suspensions, although the delay arising from the relatively long time required for self-assembly to occur (typically minutes to hours) is currently the largest obstacle in the transition from batch to continuous production of photonic CNC materials.

## 3 Polymer Mesophases for Responsive Structural Color

**Figure 4:**
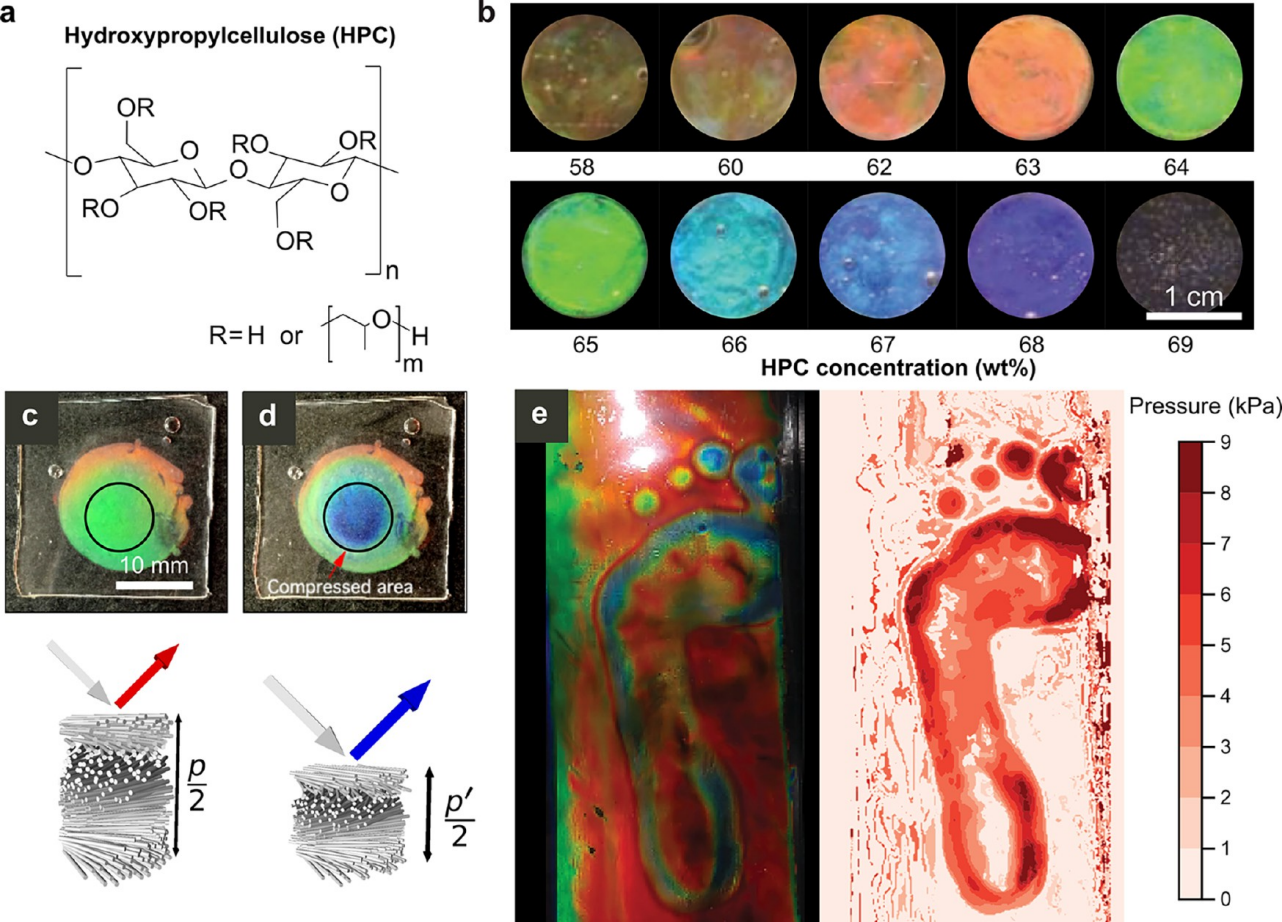
(a) The molecular structure of HPC. (b) The rainbow of colors that can be produced by tuning the concentration of HPC in water. Reproduced with permission from ref (50) under Creative Commons CC-BY. Copyright 2018 The Authors. (c,d) Images of an HPC mesophase encapsulated within polydimethylsiloxane at rest (c) and after compression of a circular region (d). The schematics below illustrate compression of a cholesteric domain, resulting in blue-shifted reflection. Reproduced with permission from ref (11) under Creative Commons CC-BY. Copyright 2016 The Authors. (e) Image of a footprint on a large-scale red HPC laminate produced using R2R deposition (left) and corresponding false color pressure map derived from the RGB values of the image (right). Reproduced with permission from ref (50) under Creative Commons CC-BY. Copyright 2018 The Authors.

Polymeric cellulose derivatives, in particular ethers and esters, are also known to self-organize into a cholesteric liquid crystal phase that can display visible, iridescent color.^45^ Among these, hydroxypropyl cellulose (HPC, Figure 4a) is of particular interest due to its ability to form a right-handed cholesteric mesophase with structural color when dissolved in a wide range of polar solvents, most notably water.^46−48^ In addition, HPC retains the edibility and biocompatibility of native cellulose, and as such is already in widespread use as a bulking agent in food products or as an excipient in the pharmaceutical industry. Together, these properties make HPC an excellent candidate for sustainable photonic materials.

The pitch of an HPC mesophase is primarily dependent upon its concentration (but can also be affected by temperature, molecular weight and degree of functionalization), and as such the reflected color can be easily tuned across the visible spectrum, spanning from near-infrared (ca. <60 wt %) through to ultraviolet wavelengths (ca. >70 wt %), as exemplified in Figure 4b.^47,49^ However, this flexibility comes at the cost of color stability. Specifically, the reflected wavelength will blue-shift into the ultraviolet region as the mesophase dries, ultimately resulting in transparent solid films. As such, to fully exploit the photonic properties of HPC, it is necessary to either inhibit the evaporation of the mesophase or to preserve its solution-state pitch into the solid state.

The simplest method to maintain the photonic response of an HPC mesophase is to encapsulate it within an impermeable matrix, thus preventing loss of solvent. Moreover, by encapsulating within a flexible film, this approach can be used to produce a colorimetric sensor, in which applied pressure and/or shear induces a spatially resolved compression of the cholesteric pitch and thus a change in color (Figure 4c,d).^11^ The relationship between the applied force and the resultant pitch change can be modeled using mechanical and geometric considerations, allowing for the applied pressure to be quantified and mapped to enable large-area, real-time pressure tracking (Figure 4e).^50^ Finally, by encapsulating HPC within an array of pixelated elastomeric chambers, the responsivity of the colorimetric sensor can be digitized, enabling 3D strain mapping.^51^

**Figure 5:**
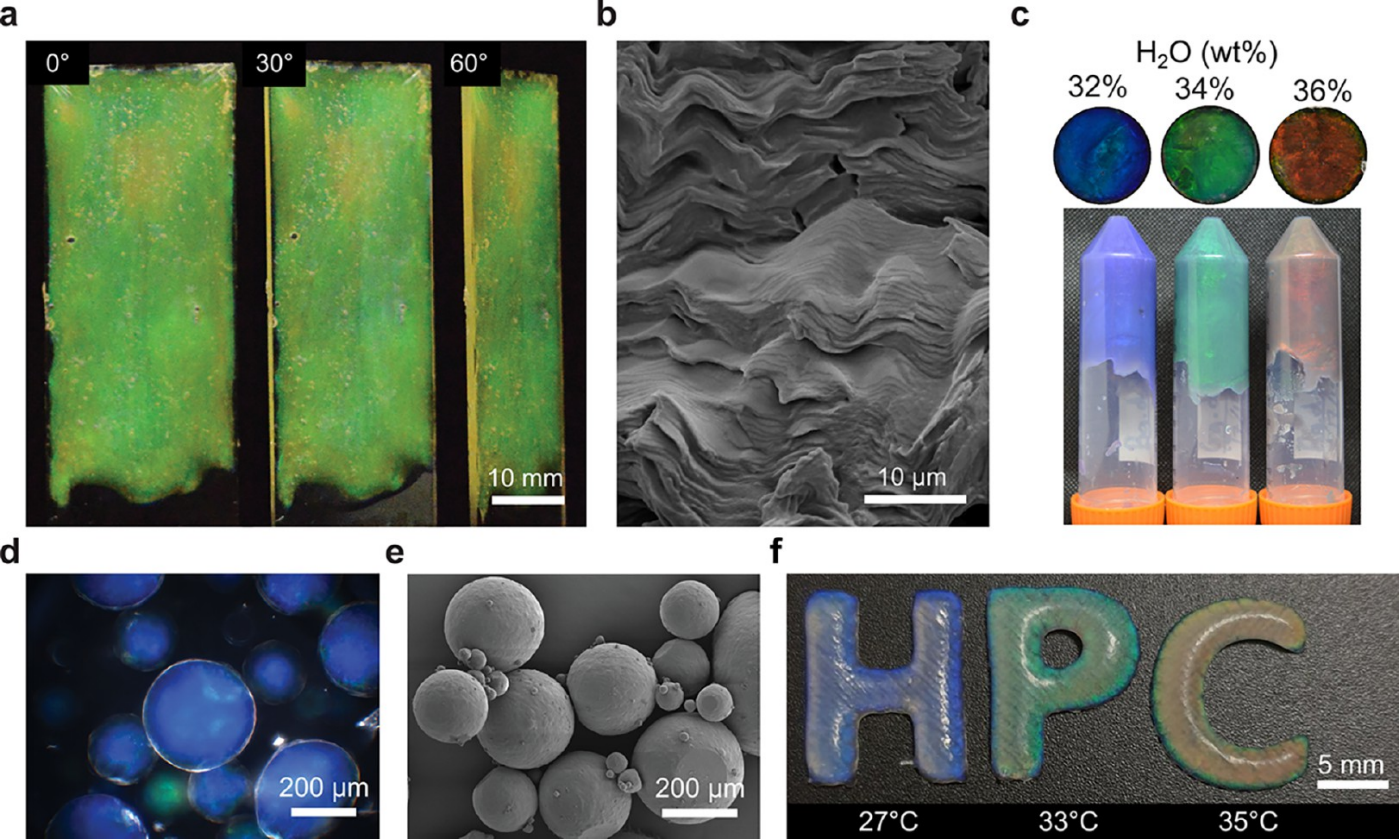
(a) Images of a cross-linked HPC film observed at different angles under diffuse illumination, demonstrating their angle-independent appearance. (b) SEM cross-section of a cross-linked HPC film, showing buckled cholesteric domains. (a,b) Reproduced with permission from ref (55) under CC-BY. Copyright 2019 The Authors. (c) Photographs of HPC gels formed with 7 wt % gelatin and increasing water content. The tubes were inverted for 48 h prior to photography. Reproduced with permission from ref (57) under CC-BY. Copyright 2021 The Authors. (d,e) Dried HPC microparticles observed by (d) bright-field optical microscopy and (e) SEM. Reproduced with permission from ref (59) under CC-BY. Copyright 2023 The Authors. (f) Photograph of 3D printed letters produced using a single feedstock of chemically functionalized HPC, but cross-linked at different temperatures. Reproduced with permission from ref (60) under CC-BY. Copyright 2022 The Authors.

While encapsulation can be used to produce responsive HPC mesophases, this approach is not suitable for applications such as colorants, where the pitch should be fixed to a specific value to achieve a consistent visual appearance. As such, many approaches have been explored to preserve the coloration of the HPC mesophase into the solid state, including chemical cross-linking,^52^ silicate composites^53^ and even irradiating with γ-rays using a nuclear reactor.^54^ These methods often perturb the HPC mesophase, which can be exploited to produce desirable optical properties. For example, drying a HPC mesophase after cross-linking with glutaraldehyde results in films with an angle-independent appearance under diffuse illumination (Figure 5a), due to localized buckling of the cholesteric domains (Figure 5b).^55^ At the same time, most current cross-linking strategies result in hard, brittle films due to the high glass transition temperature of HPC.^56^ As a consequence, many cross-linked structures lack the chromoresponsive behavior that is characteristic of HPC mesophases. However, HPC formulated with gelatin creates an interpenetrating polymer network that retains both the mechanochromic behavior of HPC and the mechanical behavior of a gelatin gel (Figure 5c).^57^ Furthermore, the recovery after compression for the HPC gel is much faster than for an encapsulated HPC mesophase, as the gelatin network suppresses shear flow and plastic deformation. Although the presence of the gelatin network increases broadband scattering, an absorbing material (e.g., carbon black) can be introduced within the sample to enhance the saturation of the color,^57^ an approach that is applicable to HPC and CNC systems in general.

Thus far, most strategies to translate the photonic properties of HPC into the solid state have resulted in continuous films or coatings. However, for practical applications, it can be advantageous to produce discrete colored particles of HPC for later use in a formulation (e.g., as colorants in paints, cosmetics, or food). To this end, it has been reported that HPC can be encapsulated into “liquid marbles” using hydrophobic silica nanoparticles as a surfactant.^58^ By controlling the loss of water into the surrounding medium, the color of these millimeter-scale HPC droplets could be tuned from blue to red. Furthermore, they were found to give a colorimetric response to changes in temperature, pressure, and solvent environment. Alternatively, to produce edible photonic pigments, an emulsified HPC mesophase can be dried at elevated temperature, resulting in solid microparticles of pure HPC with visible color (Figure 5d,e).^59^ By exploiting the thermotropic behavior of the HPC mesophase, the final pitch could be tuned solely via the drying temperature, allowing for a range of colors to be produced from a single formulation.

More generally, photonic HPC structures in arbitrary geometries can be produced by 3D printing. Through extrusion of a functionalized HPC mesophase, in combination with in situ UV cross-linking, volumetric solid-state structures can be prepared via a direct-ink writing system.^60^ Here, the color of the resultant structure can not only be tuned by varying the starting concentration of the HPC mesophase but also the temperature at which the system is cross-linked (Figure 5f). As such, printed structures of different colors can be accessed using a single feedstock. This approach has since been expanded by adding a chemical cross-linker (leading to increased buckling and a noniridescent appearance),^61^ or by mixing HPC with gelatin and a photoresponsive monomer (resulting in a printable photonic hydrogel with thermochromic behavior).^62^

## 4 Simulating the Optical Response of Complex Photonic Materials

An ideal cholesteric domain can be modeled as an optically anisotropic structure with local linear birefringence Δ*n*, within a helicoidal structure with a periodicity given by the pitch *p*. At normal incidence, the domain will selectively reflect light in a wavelength range Δλ = Δ*np* centered on a wavelength of peak reflection λ = *np*, where *n* is the average refractive index.^63,65^ In this wavelength range, circularly polarized light of the same handedness as the structure is reflected (i.e., LCP for left-handed CNC, and RCP for right-handed HPC). It is important to note that while reflection from a mirror inverts the handedness of light polarization (i.e., incident LCP is reflected as RCP and vice versa), the handedness is retained upon reflection from cholesteric structures (i.e., incident LCP is reflected as LCP). Finally, for an ideal cholesteric domain of thickness *t*, the proportion of incident unpolarized light at λ = *np* that is reflected at normal incidence is given by^63,64^
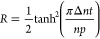
1

Notably, eq 1 illustrates that a cholesteric structure with low local birefringence will have negligible reflection; for this reason chitin nanocrystals, which can be used to produce helicoidal nanostructures with similar pitch values to CNC films, do not display visible color due to the much weaker birefringence inherited from α-chitin.^64^ Conversely, an optically thick (*t* ≫ *np*/Δ*n*) structure will reflect up to 50% of incident unpolarized light (i.e., all of one CP handedness, but not the other).

At oblique incidence of light, the central wavelength of the reflection peak λ shifts in agreement with Bragg’s law, such that
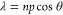
2where θ is the incident angle of light upon the cholesteric domain.^60^ Note that to calculate θ, it is necessary to include a Snell’s law correction for refraction at the air-sample interface.^26^Eq 2 shows that the iridescence of cholesteric domains arises from a blue-shift in reflection with increasing viewing angle. At grazing incidence (θ ≈ 90°), a cholesteric domain acts as a grating, and as such diffracts transmitted light, which can be used to measure the micron-scale pitch values of CNC suspensions.^42^

Real helicoidal samples deviate from ideal cholesteric structures due to the presence of multiple microscale domains with a range of orientations, and any nanoscale distortions of the local cholesteric ordering that can arise from mechanical deformations. As such, to understand the optical response of these more complex systems under a wider range of illumination conditions, it is necessary to move beyond simple analytical solutions to numerical modeling. For example, by representing helicoidal structures as a discrete stack of anisotropic layers, standard numerical approaches such as the transfer matrix (TM) method can be employed to predict the optical response.^66^ However, the scattering matrix (SM) method is computationally more robust when modeling optically thick samples, highly birefringent materials, or when illuminating at grazing incidence, as recently implemented in the open-source Python toolkit “PyLlama”.^67^

**Figure 6:**
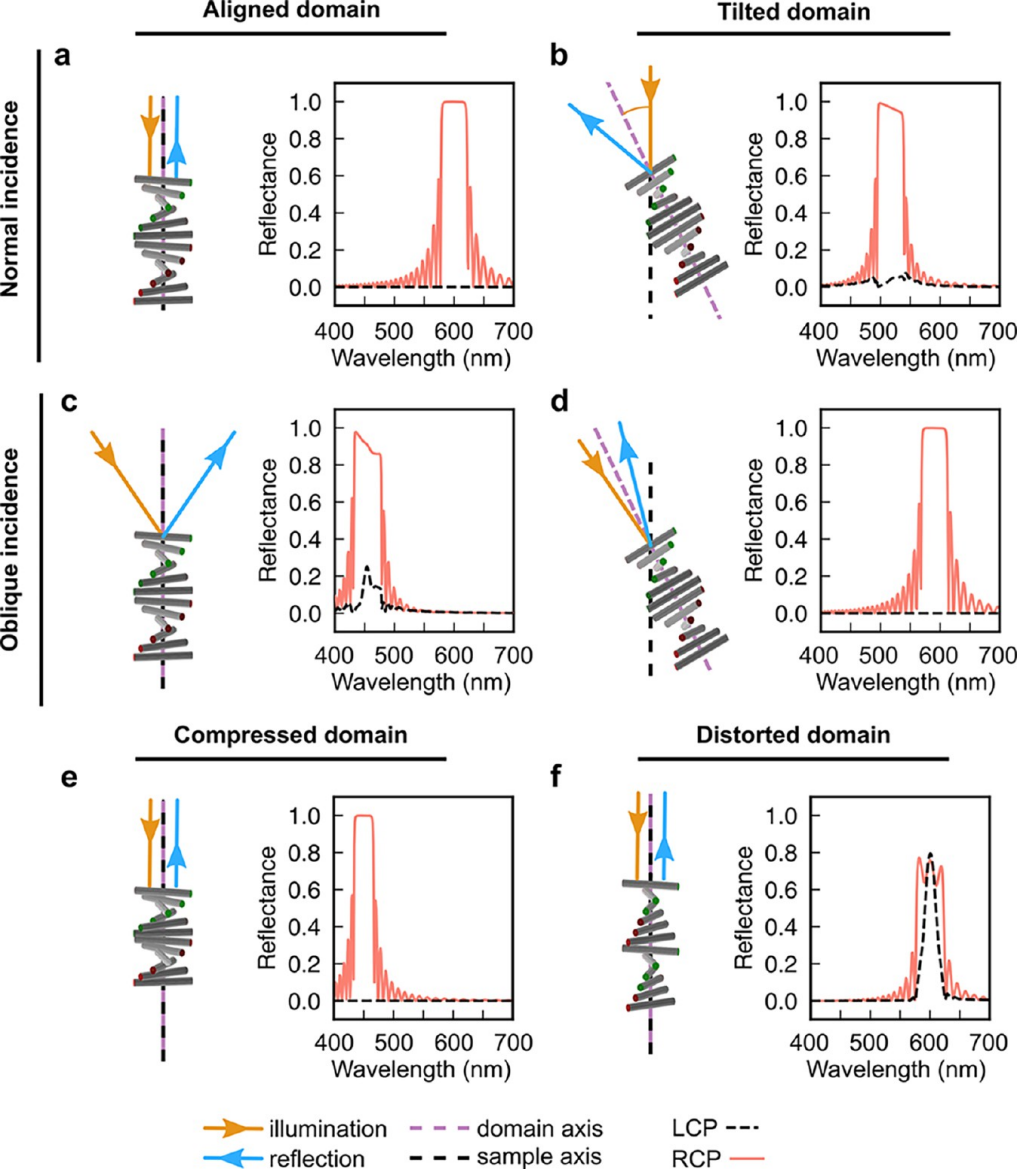
3D representations of right-handed helicoidal monodomains and associated LCP and RCP reflectance spectra modeled using PyLlama.^67^ Examples shown correspond to (a) an aligned domain illuminated at normal incidence, (b) a tilted domain at normal incidence, (c) an aligned domain at oblique incidence, (d) a tilted domain at oblique incidence, (e) an aligned domain compressed along the helix axis, and (f) an aligned domain with a distorted helicoidal structure. The sample is assumed to be a 10 μm-thick monodomain with the following parameters: *p* = 400 nm, *n* = 1.5, Δ*n* = 0.1. For simplicity, the effects of the air-sample interface (e.g., refraction) are not included in these simulations, but can be automatically included using PyLlama.

To illustrate the strengths of this approach, PyLlama was used here to model the LCP and RCP reflection spectra of right-handed helicoidal monodomains under different illumination conditions (Figure 6). By comparing these spectra, it is apparent that the angle of incidence and any distortion of the ideal cholesteric structure strongly influence the peak position, shape and degree of circular polarization. Furthermore, the physical distortion of a helicoidal structure can be mathematically related to mechanical deformations,^26^ such as compression^68^ or shear,^30^ which can be used to inform the optical multilayer model. Alternatively, simulation of the complex optical response of the sample can be used to infer plausible models of its underlying structure, as reported for the polarization independence of cross-linked HPC films.^55^

Experimental spectra often differ considerably from the predictions of monodomain models, even when taking into account the effects of distortion. For example, the width of the reflectance band often greatly exceeds the theoretical prediction (*Δλ* = *Δnp*), and optically thick samples do not reach saturation (i.e., 100% CP reflectance). These observations can be explained by expanding the numerical approach to include polydomain structures, which have discontinuities in cholesteric pitch, phase and orientation at internal boundaries. For example, this approach suggests that the characteristic banded appearance of CNC films on the microscale arises not from a horizontal domain with large pitch, but from interference at the discontinuity between stacked vertically aligned domains.^69^ Finally, while numerical modeling allows for the specular behavior of cholesteric domains to be accurately simulated, pairing it with statistical methods should allow the effects of diffusive scattering to also be included.^70^

## 5 Characterization of Structurally Colored Materials

The macroscopic appearance of photonic materials is best captured by digital photography (e.g., Figures 2f and 5a), although the illumination conditions and angle of observation must be consistent to enable comparison between samples. Another important consideration is the effect of the background or underlying substrate. While an absorbing (i.e., black) substrate eliminates unwanted back-reflection and thus enhances the contrast of the photonic response, a broadband scattering (i.e., white) substrate leads to a film displaying a complementary color at oblique angles.^38^ For polarized photography, modern (i.e., not anaglyphic) 3D cinema glasses provide an inexpensive way to differentiate between LCP and RCP light.

**Figure 7:**
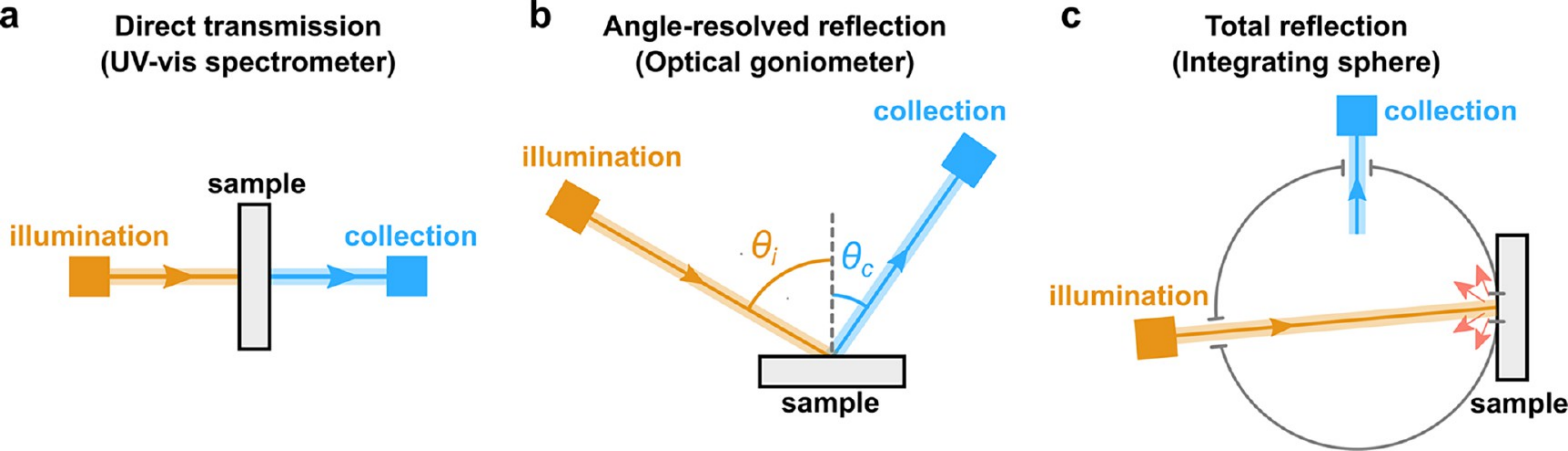
Schematic representations of three different measurement modes commonly used for macroscopic optical spectroscopy: (a) Direct ballistic transmission, measured using a UV–vis spectrophotometer, (b) angle-resolved reflection measured using an optical goniometer, and (c) total reflection measured using an integrating sphere. Note that (b) and (c) can also be used for transmission measurements by changing the configuration (not shown).

Optical spectroscopy is widely used to characterize the appearance of photonic materials in a quantitative manner. In its simplest form, the direct (ballistic) transmission of light through a sample can be measured using a commercial UV–vis spectrophotometer (Figure 7a). Circular dichroism spectroscopy combines this approach with detection of the circular polarization state of the transmitted light. However, it is important to note that CNC and HPC photonic materials are typically viewed in reflection over a range of angles, while direct transmission measurements collect only the residual complementary spectrum after all losses due to reflection and scattering have occurred.

In an optical goniometer (Figure 7b), the illumination source and detector can be independently rotated with respect to the sample. This enables, for example, measurement of the specular reflection from the surface (i.e., *θ*_*i*_ = *θ*_*c*_) across a range of illumination angles, or collection of the angular distribution of scattered light for a fixed illumination angle. Thus, while a specular scan probes the Bragg-like reflection from a well-aligned helicoidal structure, a scattering scan can reveal the degree of disorder in domain orientation, which has been proposed as a way to infer the self-assembly history of photonic CNC films.^26,68^ Conversely, an integrating sphere can be used to collect the total hemispherical reflection or transmission from a sample (Figure 7c), which has the advantage of more accurately representing the real-world appearance of the material under ambient illumination.

**Figure 8:**
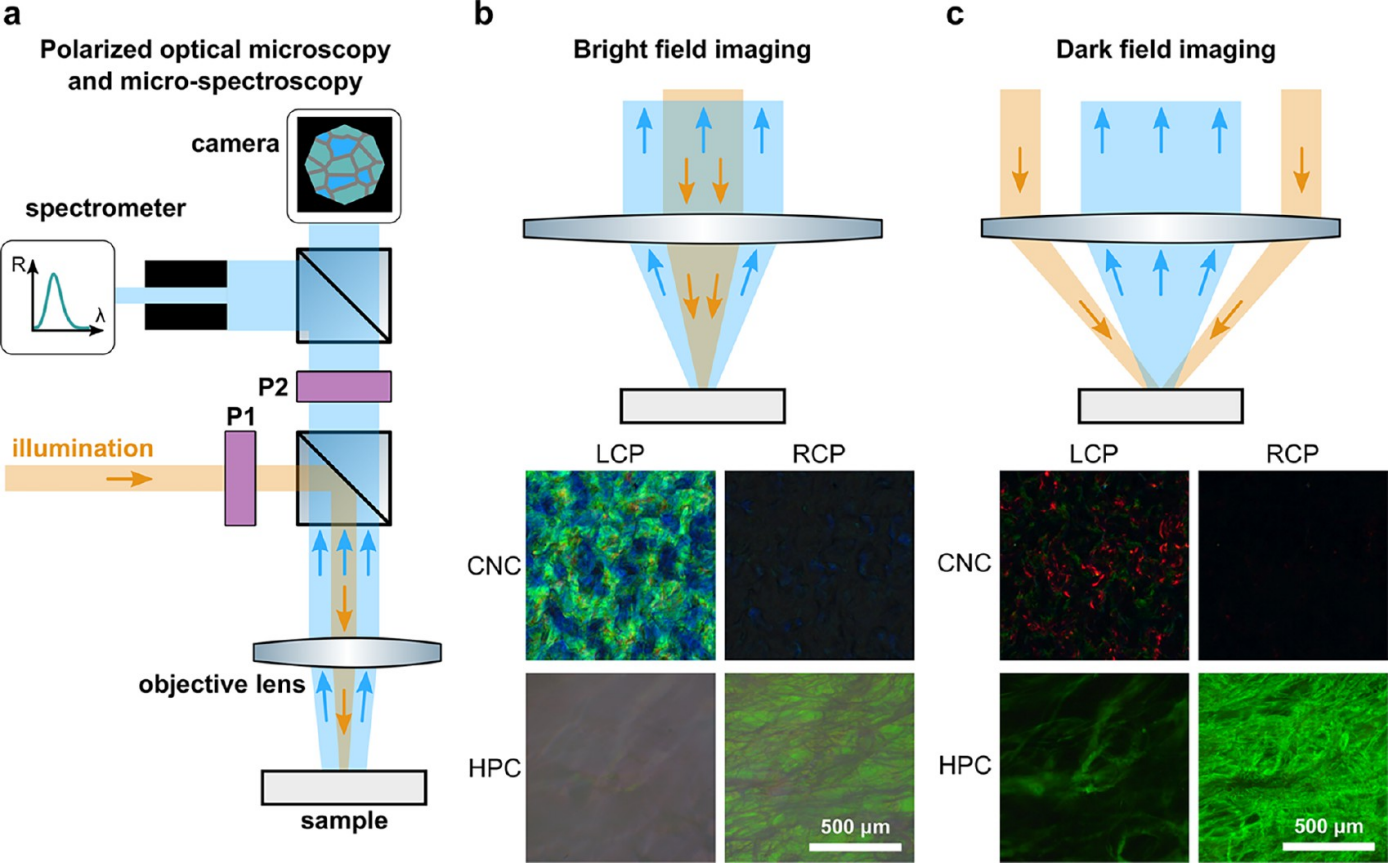
(a) Schematic of an upright optical microscope for reflection POM and microspectroscopy. The objective plays two roles, both focusing incident illumination (orange) onto the sample and collecting the reflected light (blue). P1 and P2 indicate positions for inserting additional optical components (e.g., polarizing filter, Bertrand lens). The beamsplitter allows for simultaneous acquistion of images (using a camera) and spectra (via an optical fiber-coupled spectrometer). (b,c) Schematics representing bright-field and dark-field imaging. As an example, LCP and RCP POM images are included in (b) and (c) for both a freestanding CNC film and glass-encapsulated HPC mesophase.

The microscale visual appearance of photonic materials is typically characterized by optical microscopy in bright field reflection (epi-illumination) mode (Figure 8a). The magnification of the objective lens determines the size of the region being illuminated, while its numerical aperture defines the angular cone of illumination and collection. “Bright-field” imaging is well-suited to samples with aligned helicoidal domains (Figure 8b), whereas “dark-field” imaging, in which the sample is only illuminated at higher angles of incidence, is suitable to samples with less aligned domains or when seeking to avoid interfacial reflections (Figure 8c).

Further insight is provided by polarized optical microscopy (POM), in which the incident and/or collected light is filtered for specific polarization states. For example, the broadband reflection from the film interfaces can be eliminated by viewing the sample between “crossed polarizers”. In this configuration, polarizing filters are placed orthogonally in the illumination and collection paths (P1 and P2, respectively, in Figure 8a) so that the light incident on the sample is linearly polarized and the reflected light is “analyzed” to collect only the orthogonal linear polarization component. In the particular case of helicoidal structures, the response to CP light is especially relevant (see Section 4). An LCP or RCP filter can be made by combining a linear polarizer and a quarter wave plate, which can then be used to polarize the incident light or to analyze the reflected light for a desired CP component.

The angle-resolved optical response of a sample can be probed at the microscale by introducing a Bertrand lens into the microscope beam path and imaging the back focal plane. This technique, known variously as k-space imaging, conoscopy or Fourier-plane microscopy, is complementary to macroscopic optical goniometry, and has been used to measure the local alignment of helicoidal domains in photonic CNC films.^24,69^

Microscopy can also be combined with in situ spectroscopy by coupling a fiber-optic cable confocal to the image plane (Figure 8a).^71^ This so-called microspectroscopy enables the acquisition of spectra from highly localized regions of the sample, down to the level of individual domains.^72^ As an extension to this technique, hyperspectral imaging can be achieved by performing microspectroscopy sequentially across a large area of a sample to obtain a 3D data set (i.e., *x*, *y*, λ).^69^ As a final comment on optical spectroscopy, it is important to provide the absolute reflectance or transmittance normalized to a suitable reference material (e.g., a silver mirror or a diffuse reflectance standard), rather than only providing relative values (e.g., self-normalized to the maximum value of a single spectrum or series). Furthermore, the reference material must also be measured under identical polarization conditions to the sample.

**Figure 9:**
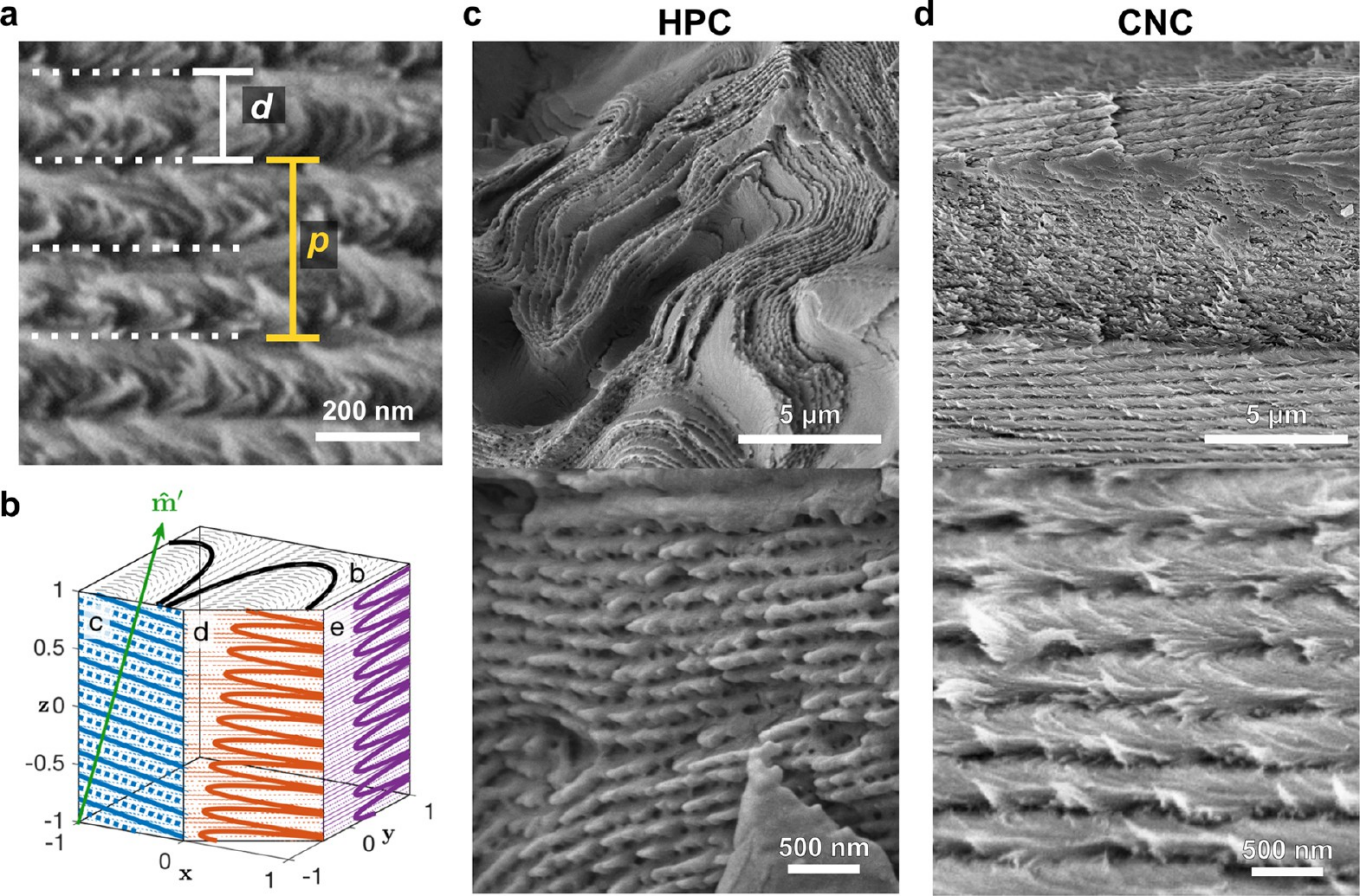
(a) Bouligand texture of a CNC film with apparent periodicity *d* (white) and apparent pitch *p* (yellow). Adapted with permission from ref (13). Copyright 2021 The Authors. (b) Schematic of the Bouligand textures expected for various fracture planes of a tilted, distorted helicoidal domain. Reproduced with permission from ref (26). Copyright 2019 American Physical Society. (c) SEM images (low and high magnification) for a green HPC microsphere showing disorder in domain orientation. Reproduced with permission from ref (59) under CC-BY. Copyright 2023 The Authors. (d) SEM images (low and high magnification) for the cross-section of a blue CNC film, showing tilted domains have larger-than-expected pitch. Reproduced with permission from ref (24) under CC-BY. Copyright 2017 The Authors.

Scanning electron microscopy (SEM), while being a nonoptical technique, can provide complementary information about the underlying nanostructure of photonic materials. If the helicoidal structure is pulled apart (rather than sliced), the exposed cross-section acquires a periodic texture known as “Bouligand arches” (e.g., Figure 9a) that are reminiscent of those first reported for twisted plywood structures in arthropods.^73^ SEM cross-sections thus enable the direct observation of the pitch and alignment of each domain within a sample. The pitch can be estimated as twice the periodicity of the Bouligand texture (*p* = 2*d*), which should correlate with the optical response using eq 2. However, the Bouligand texture and its apparent periodicity depend on the orientation of the fracture plane relative to the local helical axis (which is not readily accessible) and on any distortion of the ideal cholesteric structure (Figure 9b).^26^ While HPC and CNC samples both exhibit Bouligand textures, their SEM cross-sections reveal significant differences. HPC samples usually have low pitch variation, but with a broad distribution of domain tilts (Figure 9c). In contrast, the domains in CNC films are near-vertically aligned, but exhibit much greater variation in pitch (Figure 9d). This arises from the anisotropic compression of the cholesteric domains upon drying, which leads to larger pitch values for tilted domains.^26^ These tilted domains are the origin of the anomalous red-shifted reflection from polydomain CNC films at high incidence angles, which can be resolved by dark-field microscopy (Figure 8c) or optical goniometry.^24^ An intermediate case is observed for buckled CNC microparticles, where a considerable range of tilts and pitches are both present.^43^

## 6 Summary and Outlook

By exploiting natural cellulose and replicating the helicoidal structures found within the plant cell, a new generation of sustainable photonic pigments can be achieved. In this article we summarized two leading pathways to produce structurally colored materials from cellulose derivatives, which have the potential to not only displace traditional colorants in cosmetics and food coloration but also to unlock new visual effects (angular and polarization dependence, stimuli-responsive etc.) and applications (e.g., direct 3D printing). Lastly, while it is beyond the scope of this article, it is important to highlight that disordered nanoscale cellulose architectures are also a promising route to produce nontoxic and sustainable white pigments.^74^

With much of the cellulose photonics community focused upon developing scalable processes and exploring new applications, combined with the global expansion in CNC production and the widespread availability of HPC, there are many reasons to be optimistic about the real-world impact of these photonic materials. However, there remain several fundamental questions and technical challenges to address. For example, by elucidating the origin and mechanism of chirality transfer between individual CNCs, it may be possible to create right-handed helicoidal films, allowing for intrinsic reflection of RCP light. Furthermore, the nature of kinetic arrest and its relation to the evolution of the structure upon drying, especially buckling, also remains unclear. Greater understanding of these phenomena will enable optimization of each stage of the self-assembly process (i.e., to minimize drying time for optimal visual appearance), which is crucial for the translation to large-scale, continuous production. Finally, in contrast to model systems, the diversity inherent to naturally derived materials (i.e., CNCs: morphology, surface charge, aggregation state; HPC: degree of functionalization, molecular weight, polydispersity) makes direct comparison between studies challenging. As such, standardized protocols for characterization and data processing of both the cellulose source and the resultant photonic material need to be adopted for successful industrial scale-up.
